# Comparison of Metabolic Profiles of Fruits of *Arctium lappa*, *Arctium minus*, and *Arctium tomentosum*

**DOI:** 10.1007/s11130-024-01175-w

**Published:** 2024-04-08

**Authors:** Milan Malaník, Veronika Farková, Jitka Křížová, Alice Kresová, Karel Šmejkal, Tomáš Kašparovský, Kateřina Dadáková

**Affiliations:** 1 Department of Natural Drugs, Faculty of Pharmacy, Masaryk University, Brno, Czech Republic; 2 Department of Biochemistry, Faculty of Science, Masaryk University, Brno, Czech Republic

**Keywords:** Arctium, burdock, Metabolomics, LC-MS, Lignans, Fatty acids

## Abstract

Metabolites of the edible and medicinal plant *Arctium* have been shown to possess beneficial activities. The phytochemical profile of *Arctium lappa* is well-explored and its fruits are known to contain mainly lignans, fatty acids, and sterols. But the fruits of other *Arctium* species have not been thoroughly investigated. Therefore, this study compares the metabolic profiles of the fruits of *A. lappa*, *Arctium tomentosum*, and *Arctium minus*. Targeted metabolomics led to the putative identification of 53 metabolites in the fruit extracts, the majority of these being lignans and fatty acids. Quantification of the major lignans showed that the year of collection had a significant effect on the lignan content. Furthermore, *A. lappa* fruits contained lesser amounts of arctigenin but greater amounts of arctigenin glycoside than *A. minus* fruits. Regarding the profile of fatty acids, *A. minus* fruits differed from the others in the presence of linolelaidic acid.

**Graphical Abstract:**

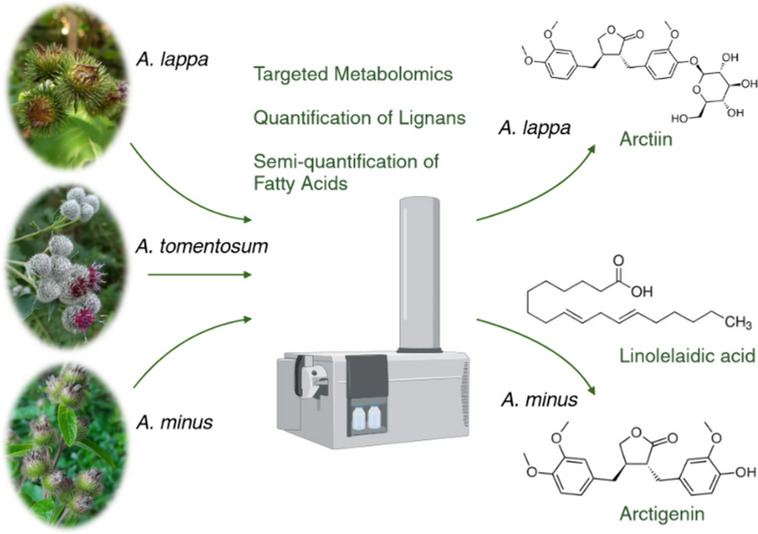

**Supplementary Information:**

The online version contains supplementary material available at 10.1007/s11130-024-01175-w.

## Introduction

*Arctium* species, commonly known as burdocks, are widely distributed all over the world. However, only four species grow in the European continent, namely *Arctium lappa* L., *Arctium minus* (Hill) Bernh., *Arctium tomentosum* Mill., and *Arctium nemorosum* Lej. Burdocks are biennial herbs growing up to 2.5 m that can be distinguished especially by leaf petioles, by the type of inflorescences, and by the presence or absence of cobweb at typical hooked involucres. *A. lappa* (greeater burdock) has cordiform leaves with solid petioles, corymbiform inflorescences, and glabrous to loosely cobwebby phyllaries. *A. minus* (common burdock) is typical with its ovate leaves with hollow petioles, racemiform inflorescences, and young phyllaries are densely cobwebby, later on glabrous. As its name suggests, for *A. tomentosum* (woolly burdock) are typical densely cobwebby phyllaries. Inflorescences are formed in a corymb and leaf petioles are solid, sometimes hollow. *A. nemorosum* has hollow petioles, racemiform inflorescences, and loosely cobwebby phyllaries. During flowering or bearing fruits, its branches are typically bent down in an arc [1, 2].

The edible roots and young leaves of *A. lappa* are frequently consumed especially in Asian countries as the main ingredients in several traditional dishes. Moreover, roots, leaves, and fruits of *A. lappa* play an integral part in traditional Chinese medicine. Extracts of different organs of *Arctium* species or compounds isolated from them are used especially in treating skin problems and possess significant antidiabetic, antioxidant, anti-inflammatory, gastroprotective, antimicrobial, and anticancer effects, as well as many other activities [3]. In particular, fruits of *A. lappa* have been extensively studied in connection with their antidiabetic activity and could therefore serve as a functional food.

More than 300 metabolites of *Arctium* species have been identified so far (reviewed in [3]). Most of these metabolites are lignans, presented primarily in the fruits and seeds of *A. lappa* and *A. tomentosum*. Another large group of metabolites is represented by specific quinic acid derivatives found almost exclusively in the roots of *Arctium* species [4], but these have also been reported in aerial parts of *A. lappa* [5] and *A. tomentosum* [6]. In addition, terpenoids and flavonoids can be found especially in burdock leaves, while fatty acids and sterols have been detected in all organs of *A. lappa* and *A. tomentosum* except the leaves. On the other hand, acetylenic compounds and saccharides are present in *Arctium* species almost exclusively in the roots [3, 6, 7]. Most of the volatile metabolites have been identified primarily in the roots of *A. lappa* [3, 8].

*A. lappa* is the most studied *Arctium* species, especially because its potential antidiabetic activity has been demonstrated both *in vitro* and *in vivo* [9], but only limited information is available for other *Arctium* species. The total phenolic content has been found to be lower in both the aerial parts and the roots of *A. tomentosum* than in those of *A. lappa* [6]. However, the highest total phenolic content was detected in extracts of seeds of *A. lappa* [10]. Several metabolites appear to be present in only some *Arctium* species, *e.g*., hyperoside has been found only in *A. tomentosum* [3, 6, 10]. The concentrations of several compounds were found to differ significantly between different organs of *A. lappa*. The highest amounts of the triterpenoids betulinic acid, oleanolic acid, and lupeol, were found in the roots of plants collected in the vegetative stage [11]. The content of bioactive compounds in a burdock depends not only on its species and the part of the plant selected, it is also influenced by environmental factors such as the temperature and precipitation the plant experiences [12].

Although the phytochemical profile of *A. lappa* has been well-investigated, other main burdocks that are widely distributed throughout Europe have, for some reason, been overlooked, and information about their content compounds is sketchy. A comparative study of the secondary metabolites found in different organs of selected *Arctium* species is, therefore, surely warranted. Presumably, *Arctium* fruits contain bioactive compounds and different species contain different contents. Therefore, this study compares the metabolic profiles of fruits of three main species growing in Europe (*A. lappa*, *A. tomentosum,* and *A. minus*) collected in the Czech Republic over two years. Targeted metabolomics based on burdock metabolites reported in the literature was used for this comparison. Furthermore, the two largest groups of metabolites found in the fruits were investigated in great detail: the major lignans in the fruits of these three burdock species were quantified and the profiles of fatty acids were compared.

## Materials and Methods

The Materials and Methods section is presented in Online Resource 1.

## Results and Discussion

*Arctium* species fruits were collected in 2021 and 2022, air-dried, and extracted. The amounts of extracts obtained per gram of the dried fruits differed significantly between the years of collection with less extract obtained in 2022. Differences in the composition of the fruit extracts might have been caused by environmental factors such as temperature, precipitation, shading, soil composition and type, soil microbiome, nitrogen content, and others [12]. At the time the samples were collected, the mean temperature in the locality was 15.3 °C in 2021 and 13.3 °C in 2022. The difference in total precipitation was much greater with only 20.2 mm in 2021 and 59.8 mm in 2022. The level of precipitation was far below normal for the time of year in 2021 [13]. The limited availability of water may have had an effect on the composition of the *Arctium* fruits, and the impact of this factor and also other factors with possible influence should be examined in detail in future experiments.

### Metabolic Profiles

Following the removal of oily compounds by two extractions into *n*-hexane, targeted-metabolomic analysis using a database of the metabolites previously found in *Arctium* species [3] led to the putative identification of 53 metabolites in burdock fruit samples (Online Resource 2). 23 of these metabolites were lignans, 10 fatty acids, 5 terpenoids, 6 were saccharides, 3 belong to the quinic acid derivatives, 4 were flavonoids, 1 was a sterol, and 1 an amino acid. This result shows that for free fatty acids the extraction into *n*-hexane was not effective. This is in accordance with Bazina & He who state that at neutral pH, more than 50% of certain free fatty acids are transferred from *n*-hexane into acetonitrile [14]. From the putatively identified metabolites, 10 were identified using MSMS spectra or standards.

**Fig. 1:**
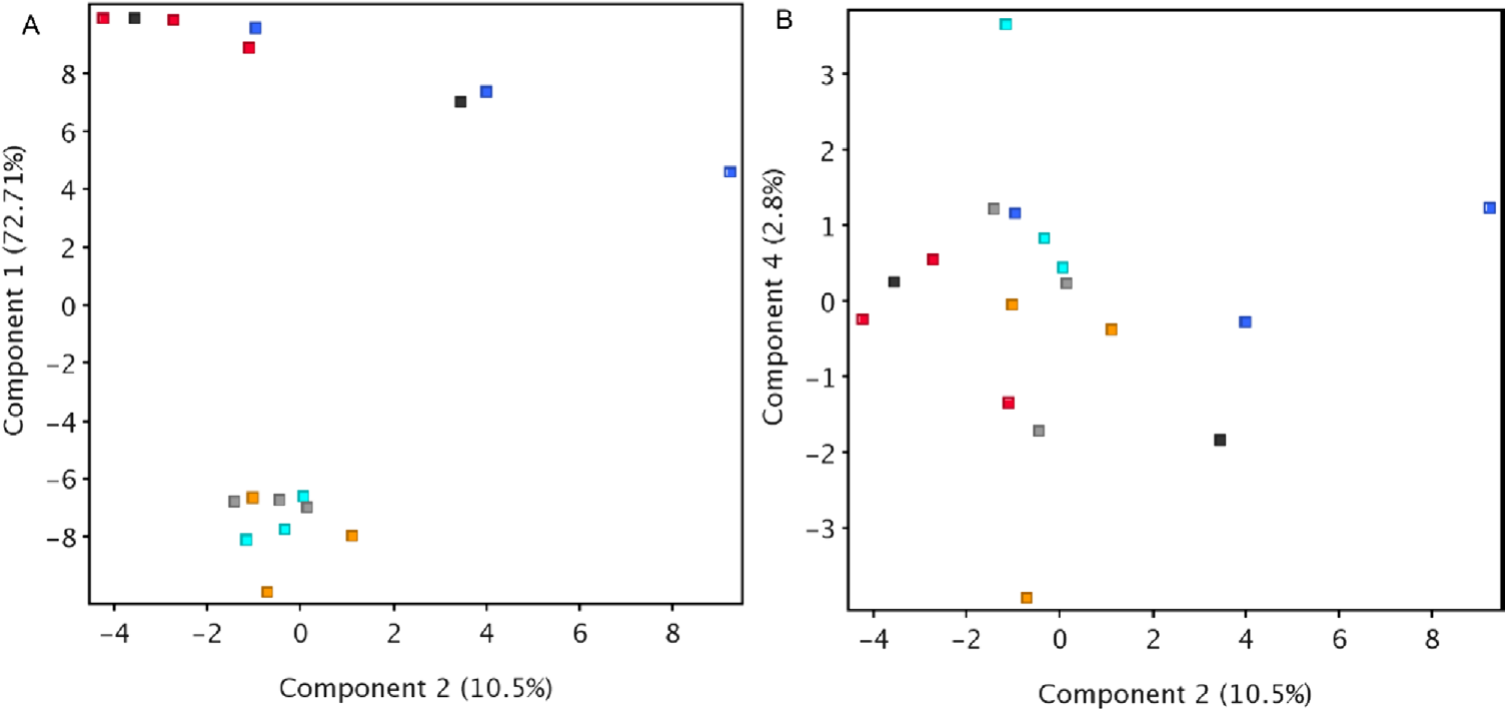
PCA analysis of *A. lappa* (samples obtained in 2021 and 2022, red and orange, respectively), *A. minus* (samples obtained in 2021 and 2022, blue and turquoise, respectively), and *A. tomentosum* (samples obtained in 2021 and 2022, dark grey and light grey, respectively). Parts A and B show plotting of components 1 and 2 and components 2 and 4, respectively

Qualitative analysis (Online Resource 2) showed that quinic acid derivatives and most flavonoids were absent in the 2022 samples. The 2022 samples also contained fewer representatives of saccharides, lignans and terpenoids. The metabolic profiles of the three species collected in the two years were visualized for comparison using PCA. Component 1, which accounted for almost 73% of the variability between the samples, separated the two years of collection (Fig. 1A). The compounds with the highest values of coefficients involved in component 1 are mostly lignans. On the other hand, the compounds with lower coefficients are fatty acids. The increase in the concentrations of flavonoids, lignans, and terpenoids could be attributed to stress caused by a lack of water in 2021, such as a drought-induced increase in carbon-based secondary metabolites that has been reported in the aerial parts of plants [15]. Lesser available water might also impact the lipid content of the fruits. A similar effect has been observed in castor beans, where water availability correlated linearly with the oil content [16].

All the species studied have a very similar profile, which is to be expected as plants from the same genus usually have similar contents. However, the *A. lappa* samples contained fewer terpenoid representatives than *A. minus* and *A. tomentosum*. Although the PCA analysis did not fully distinguish the *Arctium* species, *A. minus* showed greater values of components 2 and 4 than *A. lappa* (Fig. 1B). The compounds putatively identified as daucosterol and methyl linolenate had coefficients greater than 0.1 for components 2 and 4 and are therefore probably more abundant in *A. minus* fruits than in those of *A. lappa*. On the other hand, the compound putatively identified as arctigenin glucoside had coefficients lower than −0.1 for components 2 and 4 and is therefore probably more abundant in *A. lappa* fruits than in those of *A. minus*.

### Quantification of Lignans

**Fig. 2:**
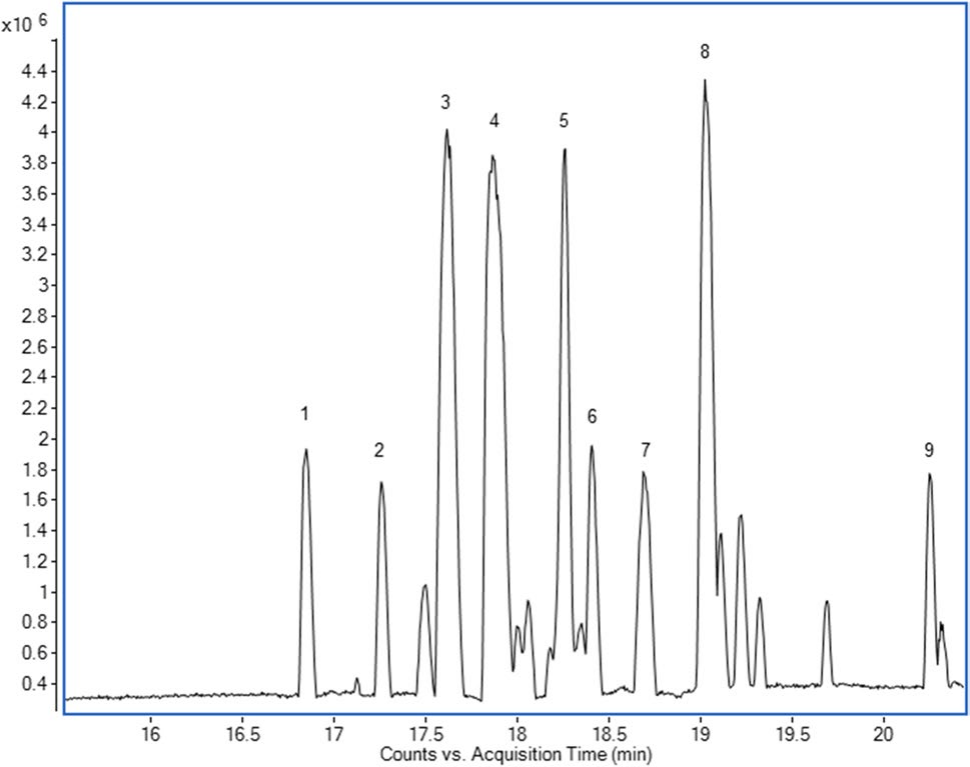
Base Peak Chromatogram of a representative sample of *A. minus* fruit extract. 1, lappaol H; 2, matairesinoside; 3, lappaol C; 4, arctiin; 5, not identified; 6, matairesinol; 7, lappaol A; 8, arctigenin; 9, diarctigenin

The major peaks in a typical base peak chromatogram of *A. minus* fruit extract were examined and those containing primarily lignan ions were identified as arctiin, arctigenin, diarctigenin, lappaol A, lappaol H, matairesinol, and matairesinoside (Fig. 2). Furthermore, the substance putatively identified as arctignan A/lappaol C/E/isolappaol C was isolated using semi-preparative HPLC and identified as lappaol C.

**Fig. 3:**
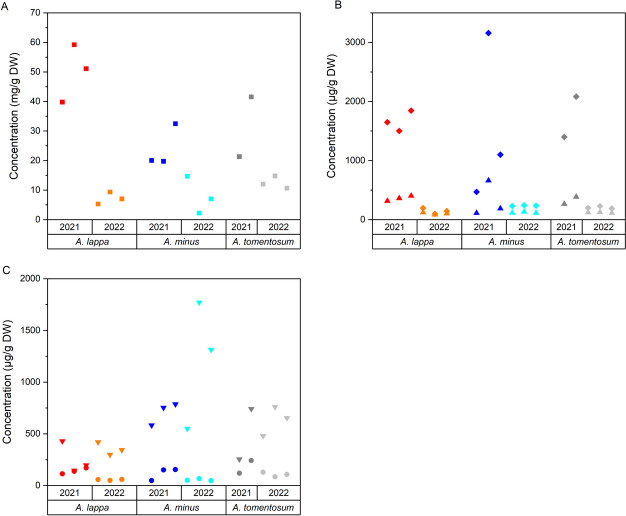
Concentrations of arctiin (**A**), the main lappaols (**B**), and other main lignans (**C**) in dried fruits of different *Arctium* species and in different years of collection. Results for *A. lappa* (samples obtained in 2021 and 2022, red and orange, respectively), *A. minus* (samples obtained in 2021 and 2022, blue and turquoise, respectively), and *A. tomentosum* (samples obtained in 2021 and 2022, dark grey and light grey, respectively), arctiin (squares), lappaol C (diamonds), lappaol H (triangles), arctigenin (inversed triangles), and matairesinoside (circles) are shown

Arctiin was quantified using an LC-DAD method with its concentration expressed in milligrams per gram of the dry weight (DW) of samples of fruits obtained from *A. lappa*, *A. minus*, and *A. tomentosum* that were collected in two different years. The effects of species and year on the concentration of arctiin were assessed (Fig. 3A). The year of collection, the species, and the combination of the year of collection plus the species were found to have statistically significant effects on the concentration of arctiin. The mean concentration of arctiin combined across the three species was 36 ± 15 mg/g DW for 2021 and only 9 ± 4 mg/g DW for 2022 (P value lower than 0.001), with this decrease from 2021 to 2022 greater for *A. lappa* than for other two *Arctium* species. When the collection from the two years were combined, a statistically significant difference in the mean concentration of arctiin was found between *A. lappa* with 29 ± 24 mg/g DW and *A. minus* with 16 ± 11 mg/g DW (P value 0.038).

An LC-Q-TOF quantification method was validated for lappaol C, lappaol H, arctigenin, and matairesinoside (Online Resource 3). These lignans were quantified in micrograms per gram of the dried fruits of *A. lappa*, *A. minus*, and *A. tomentosum* collected in two different years, and the effects of the species and the year of collection on the concentrations of the lignans were assessed (Fig. 3B, C; Online Resource 4A). The year of collection was found to have a statistically significant effect of on the concentrations of lappaol C, lappaol H, and matairesinoside with P values of 0.001, 0.006, and 0.003, respectively. The mean concentration of lappaol C was 1650 ± 780 μg/g of DW in 2021 and only 200 ± 50 μg/g of DW in 2022. The mean concentration of lappaol H was 340 ± 170 μg/g of DW in 2021 and 120 ± 10 μg/g of DW in 2022. The mean concentration of matairesinoside was 140 ± 60 μg/g of DW in 2021 and 70 ± 30 μg/g of DW in 2022. A statistically significant effect of the species on the concentration of arctigenin was also found with a mean arctigenin concentration of 310 ± 120 μg/g of DW for *A. lappa* and a much greater mean arctigenin concentration of 960 ± 480 μg/g of DW (P value 0.008) for *A. minus*. The effect of combining the year of collection with the species on the concentration of the lignans was not statistically significant.

The greater concentrations of lignans found in the samples collected in 2021 by PCA, were confirmed for arctiin and lappaol C, and also for lappaol H and matairesinoside when they were related to the dry weight of the fruits. Statistically significant differences in the concentrations of arctiin and arctigenin in the species *A. lappa* and *A. minus* were also found. For the two collection years, *A. lappa* fruits contained more of the arctigenin glycoside named arctiin and less aglycone arctigenin than those of *A. minus*. This is in accordance with the PCA analysis that indicated a greater concentration of arctigenin glucoside in *A. lappa* than in *A. minus*. These results show clearly that the fruits of *Arctium* species are very rich sources of arctiin. Although the concentration of arctiin seems to be too high, our results are otherwise in good agreement with previous reports [17, 18]. Furthermore, lappaol C and lappaol H were other most abundant compounds which is consistent with the results reported for fruits of *A. lappa* [19]. Among lignans of fruits of *A. tomentosum*, only arctiin, arctigenin, and lappaols A and F were previously identified [3]. Comprehensive studies dealing with quantification of lignans in fruits of *A. minus* and *A. tomentosum* are missing.

### Profiles of Fatty Acids

**Fig. 4:**
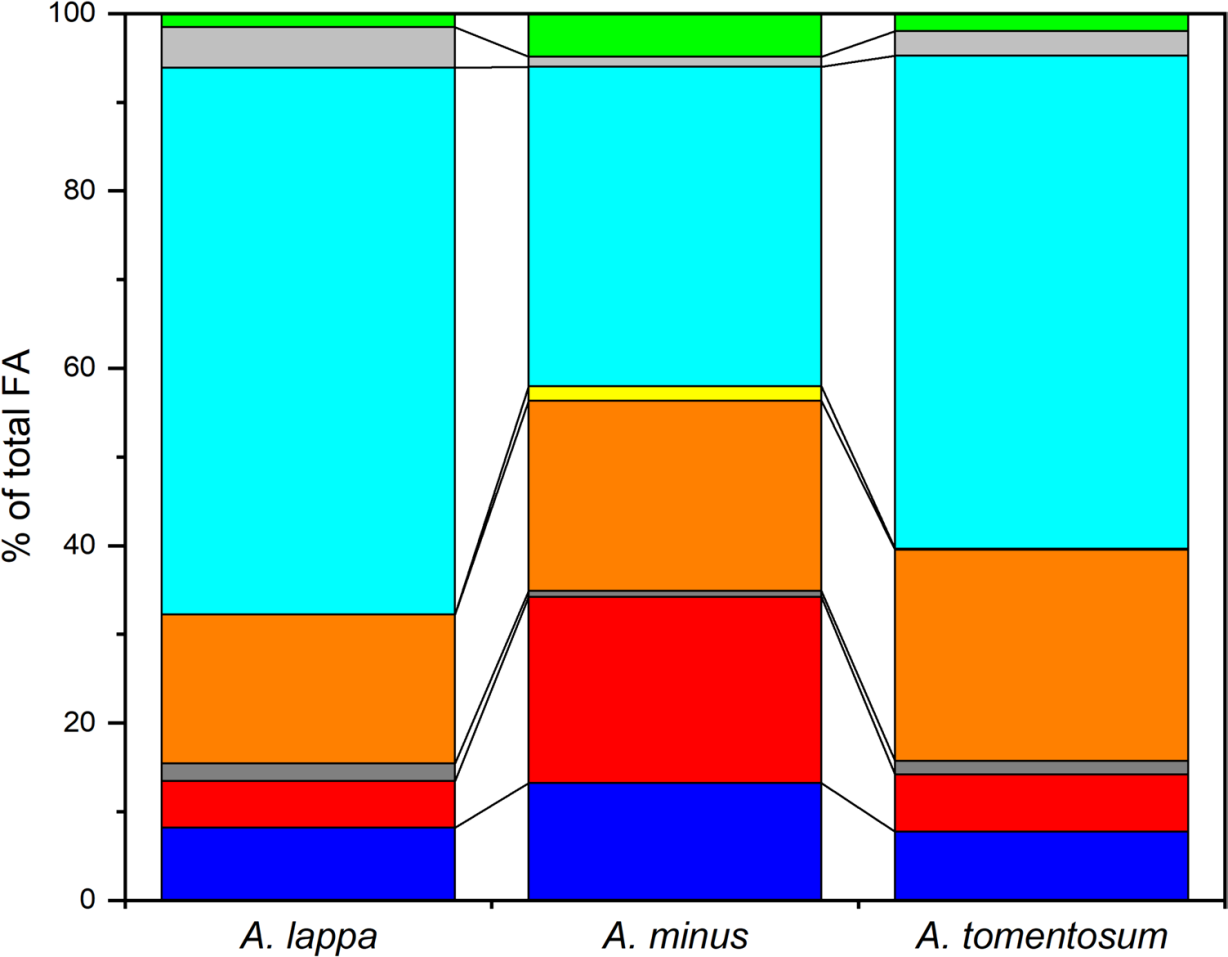
The relative contents of major fatty acids in the *n*-hexane fractions of extracts of the fruits of *A. lappa*, *A. minus*, and *A. tomentosum* collected in the year 2022. C16:0, dark blue; C16:1, red; C18:0, dark grey; C18:1n9c, orange; C18:2n6t, yellow; C18:2n6c, light blue; C18:3n6, light grey; other acids, green (ordered from the bottom to the top). Data are shown as the means of three replicates

The fatty acid compositions of the *n*-hexane fractions obtained from extracts of the three *Arctium* species for the year 2022 were compared (Online Resource 4B). The fatty acids present in the *n*-hexane fractions of *Arctium* species extracts were determined semi-quantitatively using peak areas obtained by GC-FID. The major fatty acids were palmitic (C16:0), palmitoleic (C16:1), stearic (C18:0), oleic (C18:1n9c), linolelaidic (C18:2n6t), linoleic (C18:2n6c), and gamma-linolenic (C18:3n6) acids, together representing more than 95% of the total peak area (Fig. 4). The relative content of oleic acid was significantly lower in *A. lappa* than in *A. tomentosum* (P value 0.013) and the content of gamma-linolenic acid was significantly higher in *A. lappa* than in *A. minus* (P value 0.020). Furthermore, only the extracts of *A. minus* contained the *trans*-fatty acid linolelaidic acid, a metabolite with potentially adverse effects on human health, including an increased risk of coronary heart disease and the promotion of inflammation [20, 21].

*A. lappa* fruits seemed to have a more favorable composition of fatty acids with a lower content of oleic acid than those of *A. tomentosum* and a greater content of polyunsaturated linolenic acid than those of *A. minus*. However, the difference in the linolenic acid content may have been caused by incomplete extraction of this acid from acetonitrile into *n*-hexane, as in the acetonitrile fraction subjected to metabolomic analysis, the amount of linolenate was greater in *A. minus* extracts than those of *A. lappa*, which is in contrast to the results obtained by the analysis of fatty acids. Comparison with the literature is problematic as reports dealing with the fatty acid composition of *Arctium* species fruits are scarce. Previously, the seeds of *A. lappa* and *A. minus* have been found to be rich in linoleic acid (C18:2n6c) [22, 23], which is in good agreement with our results. Interestingly, although linolelaidic acid has not been observed in *A. minus* seeds previously, Morris et al. did detect the presence of *trans*-3,*cis*-9,*cis*-12-octadecatrienoic acid [23]. Therefore, *A. minus* is potentially a source of harmful *trans* fatty acids. Unfortunately, no report of the fatty acid composition of *A. tomentosum* fruits has been found. Only one study describes the fatty acid composition of the inflorescences of *A. tomentosum* with palmitic and linoleic acid as dominant compounds [7]. However, the composition of fatty acids in these fruits can vary with time.

## Conclusions

Extracts of *Arctium* species fruits contain mainly lignans and fatty acids and the composition is greatly affected by the growing conditions in the year of fruit collection. In the year 2021 with very low total precipitation, more extract per gram of the dried fruit was obtained and it had a greater content of lignans than in 2022, a year with roughly normal total precipitation. There are also differences between *Arctium* species. *A. minus* fruits are characterized by a lower content of the major lignan arctiin, a greater content of aglycone arctigenin, and by the presence of linolelaidic acid as compared to the fruits of the most researched species *A. lappa*. Based on these results, we conclude that *Arctium* species fruits can serve as sources of bioactive compounds. However, the choice of *Arctium* species and environmental factors such as the availability of water should be considered when collecting the fruits.

### Supplementary Information

Online Resource 1 (DOCX 25.9 KB)

Online Resource 2 (XLSX 16 KB)

Online Resource 3 (XLSX 11 KB)

Online Resource 4 (XLSX 11 KB)

## Data Availability

The research data will be available on request.
