# Supplementary material for: Comparison of Metabolic Profiles of Fruits of Arctium lappa, Arctium minus, and Arctium tomentosum
Source: Plant Foods Hum Nutr. 2024 Apr 8;79(2):497–502. doi: 10.1007/s11130-024-01175-w (PMC11178601; doi:10.1007/s11130-024-01175-w)
Supplement: Supplementary file 1 — (DOCX 25.9 KB) [file 11130_2024_1175_MOESM1_ESM.docx]

Comparison of metabolic profiles of fruits of Arctium lappa, Arctium minus, and Arctium tomentosum; Plant Foods for Human Nutrition; Malanik, Farkova, Krizova, Kresova, Smejkal, Kasparovsky, and Dadakova; Masaryk University, Brno, Czech Republic; k.dadakova@mail.muni.cz

**Materials and Methods**

Plant material

Plant material was collected in several localities across the Czech Republic in September of 2021 and 2022 (Table S1). In 2021, three samples of *A. lappa*, three samples of *A. minus*, and two samples of *A. tomentosum* were collected and in 2022, three samples of each species were collected. The collected material was authenticated by Dr. Malaník (Dept. of Natural Drugs, Faculty of Pharmacy, Masaryk University, Brno) [S1, S2]. Voucher specimens of all samples have been deposited in the herbarium of the Dept. of Natural Drugs, Masaryk University, Brno.

Table S1. Species, locations, and voucher specimens of plant samples used in the study

|  | **Location** | **Voucher** | **Weight of plant material used for the extraction (g)** | **Extraction yield** |
| --- | --- | --- | --- | --- |
| *A. lappa* | N 49°14′08″, E 16°37′08″  alt.: 290 m a.s.l. | ALAPPA092021/2 | 10.019 | 1.113 |
| *A. lappa* | N 49°14′22″, E 16°33′56″  alt.: 290 m a.s.l. | ALAPPA092021/4 | 10.010 | 1.306 |
| *A. lappa* | N 49°26′58″, E 17°28′04″  alt.: 225 m a.s.l. | ALAPPA092021 | 10.001 | 1.426 |
| *A. minus* | N 49°14′48″, E 16°37′00″  alt.: 355 m a.s.l. | AMIN092021/2 | 10.012 | 1.080 |
| *A. minus* | N 49°26′49″, E 17°27′57″  alt.: 220 m a.s.l. | AMIN092021/3 | 10.052 | 1.426 |
| *A. minus* | N 49°08′45″, E 16°14′58″  alt.: 355 m a.s.l. | AMIN092021 | 9.972 | 0.825 |
| *A. tomentosum* | N 49°11′27″, E 16°12′38″  alt.: 390 m a.s.l. | ATOM092021 | 10.004 | 0.812 |
| *A. tomentosum* | N 49°26′45″, E 17°28′46″  alt.: 245 m a.s.l. | ATOM092021/3 | 10.137 | 1.364 |
| *A. lappa* | N 49°11′55″, E 16°27′59″  alt.: 355 m a.s.l. | ALAPPA092022 | 10.402 | 0.484 |
| *A. lappa* | N 49°10′04″, E 17°44′33″  alt.: 400 m a.s.l. | ALAPPA092022/2 | 16.539 | 0.483 |
| *A. lappa* | N 49°14′10″, E 16°37′12″  alt.: 290 m a.s.l. | ALAPPA092022/3 | 10.865 | 0.559 |
| *A. minus* | N 49°11′44″, E 16°28′25″  alt.: 340 m a.s.l. | AMIN092022 | 8.768 | 0.398 |
| *A. minus* | N 49°09′45″, E 16°13′56″  alt.: 390 m a.s.l. | AMIN092022/2 | 1.452 | 0.141 |
| *A. minus* | N 49°08′45″, E 16°14′58″  alt.: 355 m a.s.l. | AMIN092022/3 | 15.282 | 0.662 |
| *A. tomentosum* | N 49°10′05″, E 17°44′35″  alt.: 400 m a.s.l. | ATOM092022 | 11.377 | 0.688 |
| *A. tomentosum* | N 49°14′36″, E 16°36′58″  alt.: 325 m a.s.l. | ATOM092022/2 | 10.472 | 0.532 |
| *A. tomentosum* | N 49°11′27″, E 16°12′38″  alt.: 390 m a.s.l. | ATOM092022/3 | 6.272 | 0.430 |

The air-dried plant material (ca. 10 g) was extracted three times with 96% EtOH (1:20, *m*/*v*) at room temperature. The solvents were removed using a rotavapor and the crude extracts obtained were stored in a refrigerator for later use. Before analysis, ca. 5 mg aliquots of the extracts were dissolved in ca. 1 mL of a mixture of acetonitrile and dimethylformamide (9:1, *v*/*v*) each to obtain a concentration of 5 mg/mL (*w/*v), and extracted twice into 1 mL of *n-*hexane, to reduce the content of oily compounds. The purified samples were used for metabolomics and the *n-*hexane fractions were used for the analysis of fatty acids.

Metabolomics

Targeted-metabolomic analyses were performed using HPLC-TOF (Agilent 6545 Q-TOF, Agilent Technologies, USA). The plant extracts were diluted with 5% methanol in acidic water (0.01% acetic acid), filtered, and analyzed according to the method published earlier [S3]. Analytes were separated using a reverse-phase column (EclipsePlus C18, 2.1 × 50 mm, 1.8 µm, Agilent Technologies, USA) and gradient elution. The mobile phases used were (A), 0.01% aqueous acetic acid, and (B), methanol. The gradient conditions were as follows: 5 to 20% of B from 0 to 10 min, 20 to 100% of B from 10 to 20 min, 100% of B from 20 to 30 min, and finally, 5% of B from 30 to 40 min, all at a flow rate of 0.3 mL/min. The injection volume was 1 µL. An electrospray ion source was used under the following conditions: acquisition mode 100–1700 *m/z*, gas temperature 300 °C, gas flow 8 L/min, ion polarity negative, capillary voltage 4000 V, and fragmentor voltage 150 V. The analytes were putatively identified based on MS analysis. Following MSMS analysis of a mixed sample, some of the analytes were identified by comparing their mass spectra with the Metlin database (The Scripps Research Institute, La Jolla, CA, USA, <https://metlin.scripps.edu>).

Based on the targeted metabolomics, the lignans arctiin, arctigenin, matairesinoside, lappaol C, and lappaol H were selected for quantification. Arctiin, arctigenin, and matairesinoside standards were purchased (arctiin and arctigenin from AdooQ Bioscience and matairesinoside from ChemFaces), lappaol C and lappaol H were isolated from the samples of *A. lappa* (unpublished data). Arctiin was quantified using LC-DAD and other lignans using LC-MS.

Determination of lignans by HPLC-MS

The samples were diluted in 5% methanol and 0.1% aqueous acetic acid and purified by dispersive solid phase extraction (SPE). 100 mg of C18 sorbent was conditioned first with 1 mL of methanol and then with 1 mL of 5% methanol and 0.1% acetic acid in water. Subsequently, the sorbent was mixed with 1 ml of the diluted sample and washed with 1 mL of 10% methanol. The analytes were eluted with 1 mL of acetonitrile. Arctigenin, matairesinoside, lappaol C, and lappaol H were determined using an LC-Q-TOF instrument and identified by comparing the obtained retention times and mass spectra with those of the respective standards. The quantification method was developed and validated using a reverse-phase column (EC150/2 Nucleoshell Biphenyl, 2 × 150 mm, 2.7 µm, Macherey-Nagel, Germany) and gradient elution. The mobile phases used were (A), 0.01% aqueous acetic acid, and (B), methanol. The gradient conditions were as follows: 5% of B from 0 to 2 min, 50 to 100% of B from 2 to 12 min, 100% of B from 12 to 14 min, and finally, 5% of B from 14 to 20 min, all at a flow rate of 0.3 mL/min. The injection volume was 5 µL. An electrospray ion source was set under conditions similar to those described in Chapter 2.3 with the exception of fragmentor, which was set to 120 V. The analytes were quantified using the peak areas of their [M−H]^−^ ions (Table S2). Calibration curves were drawn using the standards in MassHunter Quantitative Analysis software (Agilent Technologies, USA).

Table S2. Mass to charge ratios of [M−H]^−^ ions of quantified lignans.

| Lignan | *m*/*z* |
| --- | --- |
| Lappaol C | 553.2079 |
| Lappaol H | 749.2815 |
| Arctigenin | 371.15 |
| Matairesinoside | 519.1872 |

Quantification of arctiin by HPLC-DAD

The levels of arctiin in samples were determined by HPLC-DAD. 5 mg of each sample were dissolved in 1 mL EtOH to obtain a concentration of 5 mg/mL (m/V). HPLC analysis was done using an Agilent 1100 instrument equipped with a Diode Array Detector (DAD) (Agilent Technologies, USA) and an Ascentis Express RP-Amide analytical column (100 mm × 2.1 mm, particle size 2.7 μm, Supelco, USA). Gradient elution with the composition of the mobile phase as follows: 10–100% MeCN and 90–0% 0.2% HCOOH, over 36 min was used. The injection volume of 1 µL was used with a flow rate of 0.3 mL/min and a column temperature of 40 °C. A calibration curve was used to quantify arctiin. Five different concentrations (0.125–1 mg/mL) were used to prepare a standard curve of arctiin constructed by a linear regression analysis. The final concentration of arctiin in each test sample was expressed as the mean of the peak areas at 280 nm of three replicates.

Fatty acid analysis

Fatty acids were profiled using derivatization and analysis by GC-FID [S5-S7]. 200 µL of the *n*-hexane fraction of each sample was mixed with 30 µL of 2M KOH in MeOH. The mixture was shaken vigorously in a stoppered test tube for 5 minutes. The test tube was allowed to rest until the upper layer of solution became clear. The upper layer containing fatty acids methyl esters (FAMEs) was then transferred to a GC vial for injection into the GC.

The FAMEs were analyzed using an Agilent 8860 (Agilent Technologies, USA) gas chromatograph with flame ionization detector (GC-FID). A capillary column (ZB-FAME, 30 m × 0.25 mm × 0.25 µm, Phenomenex, USA) was used with helium as the carrier gas at a flow rate of 1.5 mL/min, an inlet split ratio of 2:1. Exactly 5 µL was injected with a precise 10-μL syringe (Agilent Technologies, USA). The run time was 54.4 min with the oven temperature programmed as follows: initial temperature 40 °C; held for 1.0 min; temperature ramp 25 °C/min to 150 °C; held for 2 min, then temperature ramp 2 °C/min to 240 °C, held for 2 min. The injector and detector temperatures were both set at 250 °C. Identification was done using the FAMEs mix standard (Sigma-Aldrich, USA) where we were able to determine 37 FAMEs. The relative content was calculated from the sum of all found FAMEs.

Statistical analysis

Targeted-metabolomic analyses were performed using Mass Profiler Professional 15.0 software (Agilent Technologies, USA) based on the database of metabolites previously found in *Arctium* species [S4]. For principal component analysis (PCA), the data were normalized by percentile shift (75%) and baselined to the median of all samples. Differences in the lignan concentrations between the three *Arctium* species and the two years of harvesting were assessed using factorial ANOVA. Differences in the relative amounts of fatty acids between the three *Arctium* species were assessed using one-way ANOVA. The P-value cut-off for significant results was set to 0.05.

References

S1. Štěpánek J. (2004) *Arctium* L. - lopuch. In: Slavík B, Štěpánková J. Štěpánek J. (eds), Květena České republiky 7 [Flora of the Czech Republic 7]. Academia, Praha

S2. Štěpánek J (2019) *Arctium* L. - lopuch. In: Kaplan Z, Danihelka J, Chrtek J, et al. (eds) Klíč ke květeně České republiky [Key to the flora of the Czech Republic]. Academia, Praha

S3. Dadáková K, Jurasová L, Kašparovský T, et al (2021) Origin of Wine Lignans. Plant Foods Hum Nutr 76:472–477. https://doi.org/10.1007/s11130-021-00928-1

S4. Wang D, Bădărau AS, Swamy MK, et al (2019) *Arctium* Species Secondary Metabolites Chemodiversity and Bioactivities. Front Plant Sci 10:834. https://doi.org/10.3389/fpls.2019.00834

S5 Petrović M, Kezić N, Bolanča V (2010) Optimization of the GC method for routine analysis of the fatty acid profile in several food samples. Food Chemistry 122:285–291. https://doi.org/10.1016/j.foodchem.2010.02.018

S6 Zhang H, Wang Z, Liu O (2015) Development and validation of a GC–FID method for quantitative analysis of oleic acid and related fatty acids. Journal of Pharmaceutical Analysis 5:223–230. https://doi.org/10.1016/j.jpha.2015.01.005

S7 Patel MK, Mishra A, Jha B (2016) Non-targeted Metabolite Profiling and Scavenging Activity Unveil the Nutraceutical Potential of *Psyllium* (*Plantago ovata Forsk*). Front Plant Sci 7:. https://doi.org/10.3389/fpls.2016.00431
